# Exome sequencing in paediatric patients with movement disorders

**DOI:** 10.1186/s13023-021-01688-6

**Published:** 2021-01-15

**Authors:** Anna Ka-Yee Kwong, Mandy Ho-Yin Tsang, Jasmine Lee-Fong Fung, Christopher Chun-Yu Mak, Kate Lok-San Chan, Richard J. T. Rodenburg, Monkol Lek, Shushu Huang, Sander Pajusalu, Man-Mut Yau, Cheung Tsoi, Sharon Fung, Kam-Tim Liu, Che-Kwan Ma, Sheila Wong, Eric Kin-Cheong Yau, Shuk-Mui Tai, Eva Lai-Wah Fung, Nick Shun-Ping Wu, Li-Yan Tsung, Jan Smeitink, Brian Hon-Yin Chung, Cheuk-Wing Fung

**Affiliations:** 1 Department of Paediatrics and Adolescent Medicine, LKS Faculty of Medicine, The University of Hong Kong, Pok Fu Lam, Hong Kong SAR China; 2 Radboud Centre for Mitochondrial Medicine, Radboud University Nijmegen Medical Centre, Nijmegen, The Netherlands; 3 Department of Genetics, Yale School of Medicine, New Haven, USA; 4 Affiliated Hospital of Nantong University, Nantong, China; 5 The First Affiliated Hospital, Nanjing Medical University, Nanjing, China; 6 Department of Clinical Genetics, United Laboratories, Tartu University Hospital, Tartu, Estonia; 7 Department of Clinical Genetics, Institute of Clinical Medicine, University of Tartu, Tartu, Estonia; 8 Department of Paediatrics and Adolescent Medicine, Tseung Kwan O Hospital, Tseung Kwan O, Hong Kong SAR China; 9 Department of Pediatrics, Centro Hospitalar Conde de Sao Januário Hospital, Macau SAR, China; 10 Department of Paediatrics and Adolescent Medicine, Kwong Wah Hospital, Yau Ma Tei, Hong Kong SAR China; 11 Department of Paediatrics and Adolescent Medicine, Pamela Youde Nethersole Eastern Hospital, Chai Wan, Hong Kong SAR China; 12 Department of Paediatrics and Adolescent Medicine, United Christian Hospital, Kwun Tong, Hong Kong SAR China; 13 Department of Paediatrics and Adolescent Medicine, Hong Kong Children’s Hospital, Ngau Tau Kok, Hong Kong SAR China; 14 Department of Paediatrics and Adolescent Medicine, Princess Margaret Hospital, Kwai Chung, Hong Kong SAR China; 15 Department of Paediatrics, Prince of Wales Hospital, Sha Tin, Hong Kong SAR China; 16 Department of Paediatrics, Queen Elizabeth Hospital, Yau Ma Tei, Hong Kong SAR China; 17 Department of Paediatrics and Adolescent Medicine, Queen Mary Hospital, Pok Fu Lam, Hong Kong SAR China; 18 Department of Paediatrics and Adolescent Medicine, The Duchess of Kent Children’s Hospital, Pok Fu Lam, Hong Kong SAR China; 19 Department of Pediatrics, The University of Hong Kong-Shenzhen Hospital, Shenzhen, China

**Keywords:** Movement disorders, Whole exome sequencing, Genetic diagnosis, Treatment

## Abstract

**Background:**

Movement disorders are a group of heterogeneous neurological diseases including hyperkinetic disorders with unwanted excess movements and hypokinetic disorders with reduction in the degree of movements. The objective of our study is to investigate the genetic etiology of a cohort of paediatric patients with movement disorders by whole exome sequencing and to review the potential treatment implications after a genetic diagnosis.

**Results:**

We studied a cohort of 31 patients who have paediatric-onset movement disorders with unrevealing etiologies. Whole exome sequencing was performed and rare variants were interrogated for pathogenicity. Genetic diagnoses have been confirmed in 10 patients with disease-causing variants in *CTNNB1*, *SPAST*, *ATP1A3*, *PURA*, *SLC2A1*,* KMT2B*, *ACTB*, *GNAO1* and *SPG11.* 80% (8/10) of patients with genetic diagnosis have potential treatment implications and treatments have been offered to them. One patient with *KMT2B* dystonia showed clinical improvement with decrease in dystonia after receiving globus pallidus interna deep brain stimulation.

**Conclusions:**

A diagnostic yield of 32% (10/31) was reported in our cohort and this allows a better prediction of prognosis and contributes to a more effective clinical management. The study highlights the potential of implementing precision medicine in the patients.

## Background

Paediatric movement disorders (MDs) are a group of complex and heterogeneous neurological diseases including both hyperkinetic [1] and hypokinetic disorders [2]. They are presented with overlapping phenotypes and with a wide spectrum of genetic mutations causing defects in various pathophysiological pathways [3–5].

Diagnosis of childhood MDs is not straightforward. Phenotypic diagnosis only has limitations as many symptoms may have more than one underlying etiology and any particular pathophysiology can result in a complex combination of symptoms [6]]. Genetic diagnosis allows a comprehensive understanding of the underlying pathophysiology and provides specific treatment options [7–12].

Conventionally, genetic testing is done by sequential single gene Sanger sequencing. This is an ineffective method in diagnosing diseases like MDs due to its genetic heterogeneity. With the advent of next-generation sequencing (NGS), diagnosing strategies has changed to gene-panel based NGS or whole exome sequencing (WES). Neveling et al. performed a retrospective study comparing the diagnostic yield by Sanger sequencing and NGS in five different cohorts. For patients with movement disorders, the diagnostic yield is increase from 5% by Sanger sequencing only to 20% by WES with target gene panel analysis. This shows that NGS is a more superior diagnostic tool when compare to conventional Sanger sequencing [13].

The effectiveness by gene-panel based NGS study has been shown in subsequent studies. Van Egmond et al. performed a study in 61 dystonia patients with a panel of 94 genes, reaching a diagnostic yield of 14.8% [14]. Reale et al., Montaut et al. and Graziola et al. conducted three separated studies using panels with 65, 127 and 102 genes, giving a diagnostic yield of 11.3%, 22%, and 28% [15–17]. Although these three studies started in the same year (2015), the number of genes included in the analysis differs. Another study by Cordeiro et al.’s performed the study used targeted direct sequencing, targeted panel of dystonia, of epilepsy, and of cellular energetic NGS or WES. The diagnostic yield was 51%. Although they did not mention the number of genes included in each panel, from the result, the diagnoses were made majority in epilepsy panel or WES. Six diagnoses (*CAMTA1*, *CTNNB1*, *KCNA2*, *SLC13A5*, *SLC9A6*, mitochondrial ND3) would be missed as these genes were not included in other movement studies. This demonstrated WES is superior to targeted sequencing which is limited by the pre-selected gene panels that have to be frequently updated owing to discovery of new disease-associated genes [18].

In the present study, we performed WES in a cohort of 31 patients with paediatric-onset MDs to review the genetic causes and potential treatment implications. We aim to highlight the importance of genetic diagnosis in guiding a more effective clinical management of these disorders.

## Results

### Cohort description

**Fig. 1:**
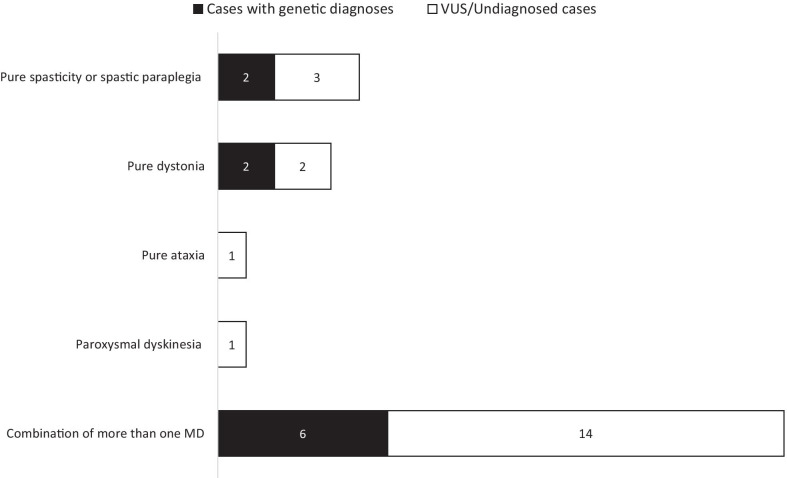
Graphical presentations of clinical and genetic outcome of patients

Clinical features of patients are summarized in Fig. 1, Table 1 and Additional file 1: Table 3. A total of 31 MDs patients were included in this study, in which 21 were males (68%) and ten were females (32%). Twenty-seven patients are Chinese (87%), while three patients (Patient 21, 22 and 30) are Pakistani and one patient (Patient 2) is African Chinese. Age of onset ranged from birth to 13 years of age. Five patients (16%) have pure spasticity or spastic paraplegia (SPG), four patients (13%) have pure dystonia, one patient (3%) has pure cerebellar ataxia, one patient (3%) has paroxysmal dyskinesia and 20 patients (65%) have a combination of more than one MDs. Dysmorphic features including microcephaly, and congenital anomalies including left atrophy kidney, duodenal atresia, pulmonary stenosis were seen in twelve patients (39%), and they are more common in patients with dystonia (9/18, 50%) when compared with other MDs patients (3/13, 23%). Abnormality in the Magnetic Resonance Imaging (MRI) of the brain were identified in fourteen patients (45%) (Table 1).

**Table 1:** Clinical features and genetic diagnosis of patients with movement disorders

Patient	Sex	Ethnicity	Movement disorders	Location	Clinical course	Onset	Dysmorphic features	Congenital anomalies	Other clinical features	Brain MRI features	aCGH findings	Variants found	Inherit- ance	ACMG classification
*Cases with pathogenic or likely pathogenic variants found*														
12	M	Chinese	Dystonia	Generalized	Non- progressive	Infancy	+	–	Mild ID, blue sclera, easy fracture	Normal	Not done	Heterozygous *CTNNB1*: c.367C > T, p.(Q123*); Heterozygous COL1A1: c.343G > T, p.(G115*) (incidental finding)	De novo	Pathogenic
14	M	Chinese	Spastic paraplegia with both upper limbs involvement	Generalized	Progressive	Infancy	–	–	–	Periventricular white matter change	Not done	Heterozygous *SPAST*: c.1253_1255delAAG, p.(Glu418del)	De novo	Pathogenic
17	M	Chinese	Dystonia, Choreoathetosis with status dystonicus	Generalized	Progressive	Infancy	–	–	Mild to moderate ID, Rhabdomyolysis	Normal	Not done	Heterozygous *GNAO1*: c.625C > T, p.(Arg209Cys)	Parents’ DNA not available	Likely pathogenic
19	M	Chinese	Spastic paraplegia	Lower limbs	Progressive	Early child- hood	–	–	Attention deficit hyperactivity disorder	Periventricular white matter changes with corpus callosum thinning	Not done	Compound heterozygous *SPG11*: c.4462_4463del, p.(V1488fs) & c.1569G > A, p.(W523*)	Paternal & Maternal	Likely pathogenic
20	M	Chinese	Rigidity with parkinsonism features, Spasticity, Paroxysmal worsening of parkinsonism	Generalized	Non- progressive	Birth	–	Pulmonary stenosis	Obstructive Sleep Apnoea Syndrome, Severe ID, laryngomalacia	Progressive cerebellar atrophy	Not done	Heterozygous *ATP1A3*: c.954C > G, p.(Ile318Met)	De novo	Pathogenic
23	M	Chinese	Dystonia	Generalized	Non- progressive	Infancy	+	–	Severe ID, Epilepsy	Normal	Normal	Heterozygous *PURA*: c.783C > G, p.(Tyr261*)	De novo	Pathogenic
27	M	Chinese	Cerebellar ataxia, spasticity	Generalized	Non- progressive	Infancy	–	–	Reactive hypoglycaemia, Mild ID	Normal	Not done	Heterozygous *SLC2A1*: c.388G > C, p.(Gly130Arg) #	De novo	Pathogenic
29	M	Chinese	Dystonia, Spasticity	Generalized	Progressive	6y	–	Left atrophic kidney	Fatty liver, Recurrent patellar dislocation, prominent capillaries	Normal	Not done	Heterozygous *KMT2B*: c.2425C > T, p.(Gln809*)	De novo	Pathogenic
30	F	Pakistani	Cerebellar ataxia, spasticity, rigidity	Generalized	Progressive	13y	–	–	Neuromuscular weakness	Periventricular white matter changes with corpus callosum thinning	Not done	Homozygous *SPG11*: c.5399_5402delinsTGGAGGAG:p.(Gln1800fs)	Paternal & Maternal	Likely pathogenic
31	M	Chinese	Dystonia, spasticity	Generalized	Progressive	Infancy	–	–	Bilateral hearing impairment, autism spectrum disorder, learning problem (formal IQ not available)	Normal	Not done	Heterozygous *ACTB:* c.547C > T:p.(Arg183Trp)	De novo	Pathogenic
*Cases with variants of unknown significance (VUS) found*														
1	M	Chinese	Cerebellar ataxia	Generalized	Non-progressive	6y	–	–	Limited intelligence with dementia, bipolar affective disorder	Stable cerebellar atrophy	Not done	Heterozygous *KCND3*: c.1917C > A, p.(Asn639Lys)	Maternal	VUS
6	M	Chinese	Dystonia, Spasticity	Generalized	Non-progressive	Birth	+	–	Mild ID, severe intrauterine growth retardation, autism spectrum disorder	Right parietal lobe developmental venous anomaly	Normal	Compound heterozygous *VPS13D*: c.5300C > T, p.(Thr1767Ile) & c.8213A > C, p.(Gln2738Pro)	Paternal & Maternal	VUS
7	F	Chinese	Dystonia, Spasticity	Generalized	Non-progressive	Birth	+	–	Mild ID to limited intelligence, intrauterine growth retardation	Dysgenesis of corpus callosum	Not done	Compound heterozygous *VPS13D*: c.5300C > T, p.(Thr1767Ile) & c.8213A > C, p.(Gln2738Pro)	Paternal & Maternal	VUS
8	F	Chinese	Cerebellar ataxia, Spasticity	Generalized	Non-progressive	Infancy	–	–	Mild ID with dementia, Ichthyosis	Progressive cerebellar atrophy	Not done	Heterozygous *KCNC3*: c.2105G > T, p.(Ser702Ile)	Paternal	VUS

*MRI*  Magnetic resonance imaging; *ACMG* American College of Medical Genetics, *VUS* variant of unknown significance, *y* years, *ID* intellectual disability; *IQ* intelligence quotient

^#^The family declined lumbar puncture

### Diagnostic yield and genetic variants found

Singleton exome was performed in 19 subjects and trio exome was performed in 10 families. Patient 17 was initially recruited for WES, unfortunately his DNA was insufficient to proceed and he passed away suddenly. Sanger sequencing of the exons and splice junctions of the *GNAO1* gene was performed for this patient due to a strong clinical suspicion for *GNAO1* defect.

Genetic diagnoses were made in 10 patients (32%), among eight were by virtual gene panels analysis and two by open-exome analysis. Disease-causing variants were found in two patients with SPG (2/5, 40%) in *SPAST* and *SPG11*, six patients with have a combination of more than one MDs (6/20, 30%) in *GNAO1*, *SLC2A1*, *KMT2B*, *SPG11, ATP1A3* and *ACTB*. The diagnosis made in two patients with pure dystonia (2/4, 50%) were by open exome analysis, one with *PURA* and one with *CTNNB1* and *COL1A1* representing one man two diseases*.* Four patients have variants of uncertain significance (VUS) associated with the phenotypes (Table 1, Fig. 1). Interestingly, patient 27 with a *SLC2A1* mutation causing glucose transporter type 1 (GLUT1) deficiency syndrome, was initially suspected to have a neurometabolic disorder due to the presence of systemic hypoglycaemia, cerebellar ataxia, spasticity and mild intellectual disability. Subsequent endocrinological workup confirmed reactive hypoglycaemia. As the family refused lumbar puncture, GLUT1 deficiency was only diagnosed after WES.

**Fig. 2:**
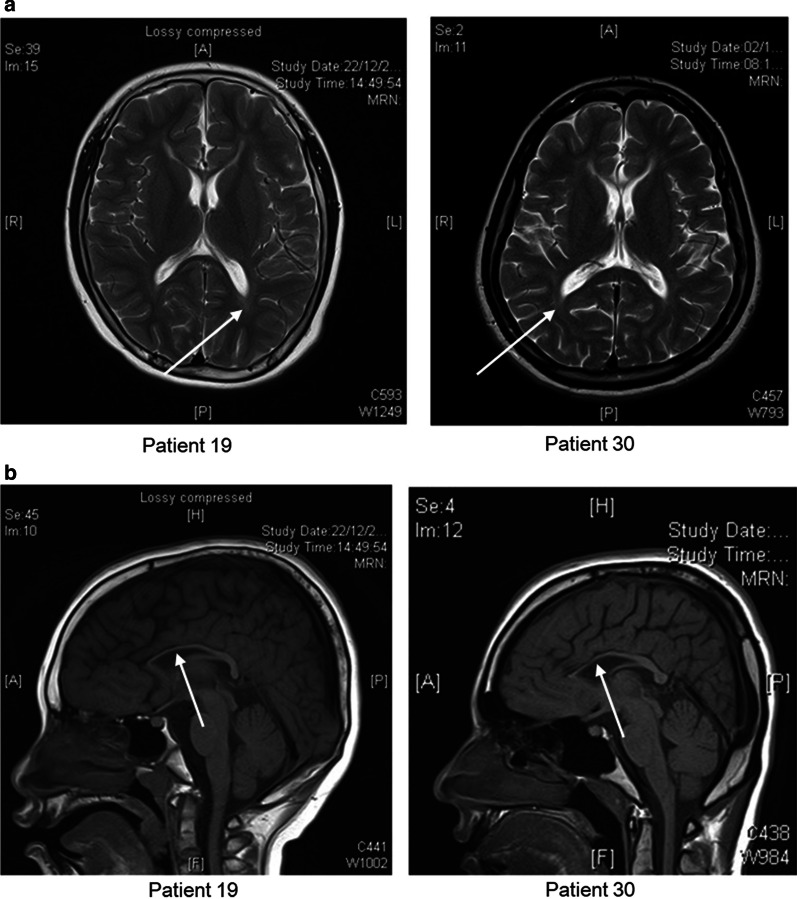
Brain Magnetic Resonance Imaging (MRI) of 2 patients with variants identified in *SPG11*. **a** Brain MRI of Patient 19 and 30 with periventricular white matter changes; **b** brain MRI of Patient 19 and 30 with thinning of corpus callosum. Arrows indicated the area with periventricular white matter changes and thinning of corpus callosum

Among the 14 patients with abnormalities in neuroimaging, 4 (29%) had genetic causes identified. Neuroimaging for patients with *SPG11* variants showed periventricular white matter changes and thinning of corpus callosum indicating that these features could be typical for *SPG11* deficiency (Fig. 2).

### Clinical follow up with treatment implications

Among the 10 genetically diagnosed patients, 8 patients (80%) have potential treatment implications (Table 2). Patient 29 with *KMT2B* mutation received globus pallidus interna deep brain stimulation (GPi-DBS) with mild improvement in dystonia a few months after the surgery and more definitive effectiveness will be evaluated in the future. Patient 17 with *GNAO1* mutation prescribed tetrabenazine and has been useful in controlling the significant dyskinesia before he passed away suddenly. Patient 30 with *SPG11* did not show any response to L-dopa. For patient 19 with *SPG11* and patient 31 *ACTB* mutation, Dopa and GPi-DBS was just started and planned, monitoring is required for treatment response. For patient 12 with *CTNNB1* and *COL1A1* mutation, Dopa was just started, and he has been referred to the endocrinologist for further management. Patient 20 and 27 decline the treatment offers.

**Table 2:** Clinical management and Genotype-targeted treatment implications of patients with variants identified

Patient	Movement disorders	Gene with variants found	Genotype-targeted treatment implication in literature	Treatment offered and clinical outcome/ follow up
12	Dystonia	*CTNNB1* and *COL1A1*	(1) *CTNNB1*: L-dopa treatment [12] (2) *COL1A1:* Treatment to prevent bone fracture resulted from osteogenesis imperfecta	Dopa treatment was offered but has not started yet Genetic finding explained the phenotypes of blue sclera and bone fractures in that patient who has been referred to the endocrinologist for further management
17	Dystonia, Choreoathetosis with status dystonicus	*GNAO1*	Tetrabenazine, GPi-DBS [9]	Tetrabenazine was used with improvement before the patient passed away with sudden death
19	Spastic paraplegia	*SPG11*	L-dopa and sapropterin treatment [8]	The patient had just commenced on Dopa treatment
20	Rigidity with parkinsonism features, Spasticity, Paroxysmal worsening of parkinsonism	*ATP1A3*	Calcium channel blockers, ATP supplementation [26]	The treatment has been offered but declined by the patient
27	Cerebellar ataxia, spasticity	*SLC2A1*	Ketogenic diets [27, 28]	The diet has been offered but the patient refused due to anticipated poor compliance
29	Dystonia, Spasticity	*KMT2B*	GPi-DBS [10, 30]	The patient has received GPi-DBS with mild improvement in dystonia a few months after the surgery and more definitive effectiveness will be evaluated in the future
30	Cerebellar ataxia, spasticity, rigidity	*SPG11*	L-dopa and sapropterin treatment [8]	The patient has been checked to have low HVA in CSF and received Dopa replacement therapy without any clinical response
31	Dystonia, spasticity	*ACTB*	GPi-DBS [31–33]	Plan has been made for the patient to be evaluated for GPi-DBS

*GPi-DBS*  Globus pallidus interna deep brain stimulation, *HVA* homovanillic acid, *CSF* cerebrospinal fluid

## Discussion

Our study includes paediatric-onset movement disorders with unrevealing etiologies after comprehensive investigations. Previous studies have investigated the genetic landscapes in cohorts with both adult and paediatric patients, or paediatric only by targeted NGS and/or WES [13–17, 19]. Diagnostic rates were relatively higher in cohorts with only paediatric patients and with the use of WES. Comparing with studies using targeted NGS approach with a diagnostic rate range from 11 to 28%, our study and Cordeiro et al.[19] with WES has a higher diagnostic rate of 32% and 51% respectively. Further looking into the diagnoses, two (2/10, 20%) and six (6/26, 23%) in our cohort and Cordeiro et al.’s cohort can be made by WES only. This shows WES is useful in making additional diagnoses in MDs patients (Table 3). Moreover, the inclusion criteria in this study are more stringent than the previous paediatric studies as patients with a clear neurometabolic phenotype which are subsequently confirmed with targeted gene sequencing were excluded. This illustrates that the diagnostic yield through WES is still considerable (32% in our study) after an initial comprehensive neurometabolic investigations.

**Table 3:** Previous studies investigating underlying genetic causes in patient cohorts with movement disorders

	Neveling et al. [13]	Van Egmond et al. [14]	Reale et al. [17]	Montaut et al. [16]	Cordeiro et al. [19]	Graziola et al. [15]	The present study
Country of patient recruitment	The Netherlands	The Netherlands	Italy	France, Luxembourg and Algeria	Canada	Italy	Hong Kong SAR, China
No. of patients with MDs	50	61 (all with dystonia)	221	378	51	148	31
Onset age	Adult and paediatric	Adult and paediatric	Adult and paediatric	Adult and paediatric	Paediatric	Paediatric	Paediatric
No. of young-onset MDs	Not specified	44	Not specified	Not specified	51	148	31
Sequencing methods	Whole exome sequencing and target data analysis	Next generation sequencing and gene panel analysis	Targeted next generation sequencing	Targeted next generation sequencing	Targeted direct, targeted next generation or whole exome sequencing	Targeted next generation sequencing	Whole exome sequencing with both targeted and exome wide analysis. One variant was identified by Sanger sequencing
No. of genes in panel	151	94	65	127	–	102	–
Diagnostic yield	20% (10/50)	14.8% (9/61)	11.31% (25/221)	22% (83/378)	51% (26/51)	28% (42/148)	32% (10/31)
Treatment implications	–	–	–	–	38% of patients with a genetic diagnosis	–	80% of patients with genetic diagnosis

### Better prediction of clinical courses

Genetic diagnoses allow the prediction of the subsequent clinical course. There is no definite difference in the clinical phenotype between the molecular positive and negative cases. Patient 14 and 17 have been initially diagnosed as cerebral palsy which is a static condition with non-progressive damage to the brain. Their neurological signs could have been overlooked if the clinical follow up was not over a prolonged period of time in terms of years. Patient 14 gradually developed progressive functional deterioration since 3 years of age from walking independently to requiring aids over 5 years with prominent spasticity over both lower limbs but minimally at the upper limbs. The neuroimaging was misleading due to the presence of periventricular leukomalacia which could be mistaken as perinatal insult. Patient 17 had an initially static course with mild generalized dystonia which evolved into recurrent status dystonicus and sudden death at 15 years old. Identification of the genetic etiologies in these patients directed more accurate predictions of the clinical course and prognosis including progressive lower limb spasticity for *SPAST* mutations and progressive MDs for *GNAO1* encephalopathy with potential treatment implication according to previous case studies [20–24].

### Potential genotype-targeted treatment implications

Treatments targeting specific genetic etiologies are significantly beneficial to patients’ prognosis. In our exome-positive cases, 80% (8/10) have potential treatment implications.

Five patients (with variants in *SPG11, CTNNB1, GNAO1, ATP1A3* and *SLC2A1*) might be managed by conventional medical and / or surgical treatments. Previous studies in patients with spastic paraplegia 11 (SPG11) demonstrated neurotransmitter abnormalities in dopamine and tetrahydrobiopterin pathways [8]. In that study, all patients responded partially to L-dopa/carbidopa and sapropterin treatment and they suggested a trial of L-dopa/carbidopa and sapropterin treatment for extrapyramidal signs and symptoms of SPG11 even with normal neurotransmitter levels [8]. However, Patient 30 with SPG11 did not show any response to L-dopa despite the presence of secondary neurotransmitter deficiency in homovanillic acid before the molecular diagnosis was made. For *CTNNB1* mutation, a recent case study reported a significant response to L-dopa treatment in a dystonic patient with a normal CSF neurotransmitter profile [12]. This response was possibly related to synaptic dopamine increase as a previous study suggested the role of beta-catenin in dopamine neurons development [25]. Treatment will be started for Patient 12. For *GNAO1* encephalopathy, tetrabenazine was demonstrated to be the most effective drug for the management of involuntary movements [9]. This drug was useful in controlling the significant dyskinesia of Patient 17 before the molecular diagnosis was confirmed and he passed away suddenly. For *ATP1A3*-associated disorders, apart from the effective symptomatic treatment by calcium channel blockers, a recent study showed that adenosine-5′-triphosphate (ATP) supplementation in an alternating hemiplegia of childhood (AHC) patient had marked improvement in AHC episodes and psychomotor development [26]. Treatment was declined by Patient 20 with paroxysmal worsening of his parkinsonism features. In addition to medical management, ketogenic diet (KD) was proved to be a very effective therapy as first line treatment for GLUT1 deficiency syndrome, which is associated with *SLC2A1* mutation, and should be started in early disease stage [27, 28]. It is a high-fat diet that produces ketone bodies serving as an alternative energy source for brain metabolism and bypassing the GLUT defect [28, 29]. It was reported that KD could help in development and restore mental decline [29]. Unfortunately, KD was declined by Patient 27 due to anticipated poor compliance to the diet.

Three out of 10 patients (with *GNAO1, ACTB* and *KMT2B* variants) could be considered for surgical interventions when medical therapies for dystonia fail. Patients with *KMT2B*-dystonia in previous studies showed good responses clinically after undergoing GPi-DBS especially for paediatric and adolescent patients [10, 30]. Patient 29 with *KMT2B* mutation, being medically intractable with 4 anti-dystonic medications (gabapentin, clonazepam, carbamazepine, trihexyphenidyl), received GPi-DBS after confirming the molecular diagnosis with mild improvement in dystonia a few months after the surgery and more definitive effectiveness will be further evaluated in the future. GPi-DBS was also demonstrated to have beneficial effect in *ACTB*-associated dystonia-deafness syndrome. In previous studies, four patients with the same mutation (p.Arg183Trp) showed substantial clinical improvement after GPi-DBS [31–33]. Therefore, patient 31 with *ACTB* mutation will be planned for GPi-DBS as the dystonia fails to improve on 5 medications (baclofen, trihexyphenidyl, L-dopa, clonazepam, gabapentin). For *GNAO1* encephalopathy, although tetrabenazine was demonstrated to be the most effective drug, emergency GPi-DBS was shown to be helpful for those patients with hyperkinetic exacerbations [9]. Furthermore, dissection of phenotype-genotype correlation suggested that different *GNAO1* mutations affect the G protein function for the signaling loop in distinct ways that implicated different treatment options [11]. This suggested possible application of precision medicine for different *GNAO1* variants identified in the patients.

Most of the previous studies in MDs patient cohorts had not investigated the therapeutic potentials based on the genetic diagnosis. Only the study of Cordeiro et al. [19] suggested that 38% of their patients with genetic diagnoses had treatment implications (Table 3), while our study has a much higher percentage of 80%. With the growth of new treatment strategies emerging in recent years, genetic testing becomes even more crucial to direct genotype-targeted therapies in MDs.

## Conclusions

Given the diagnostic yield of 32% in our patient cohort and clinical treatment implications in 80% of the molecularly diagnosed cases, WES is a valuable tool for molecular investigation in paediatric-onset MDs with unrevealing, comprehensive neurometabolic workup especially aiming for potentially treatable inborn metabolic diseases. This study demonstrated that identification of genetic etiologies of MDs allows a more accurate prediction of clinical course and guides the use of potential therapies for better clinical outcomes. As such, there is an increasing potential to develop precision medicine for treatment of MDs.

## Methods

### Patient cohort

The study was conducted in Queen Mary Hospital and Duchess of Kent Children’s Hospital, two affiliated hospitals of The University of Hong Kong (HKU). Over 4 years (2016–2019), 140 patients who were followed up longitudinally in a specialized and tertiary neurometabolic / movement disorder clinic were examined. The inclusion criterion was the diagnosis of a paediatric-onset (≤ 18 years of age) MD or combination of MDs including chorea, athetosis, dystonia, tremor, myoclonus, parkinsonism, cerebellar ataxia and spasticity as the main clinical sign(s) with unrevealing etiologies after extensive investigations. Such investigations included neuroimaging studies (Magnetic Resonance Imaging of the brain) and neurometabolic workup such as blood for lactate, gas, ammonia, amino acid, acylcarnitine profile, cholestanol, creatine, guanidinoacetate, lipid profile, vacuolated lymphocytes, lysosomal enzymes, biotinidase, copper, caeruloplasmin, very long chain fatty acids, pristanic and phytanic acids, vitamin E, total homocysteine, manganese, urate, iron profile; and urine for amino acid, organic acid, creatine, guanidinoacetate, purine and pyrimidines, oligosaccharides and glycosaminoglycans. Cerebrospinal fluid (CSF) for routine microscopy, glucose, protein, amino acids, lactate and neurotransmitter profiling were performed in 23 patients (74%). The reasons for not performing lumbar puncture include decline by the families or the results of WES were already available. Targeted gene sequencing was performed for patients with a clearly abnormal biochemical and / or radiological phenotype suggestive of a neurometabolic disorder. The exclusion criterion was disorders with acquired or other secondary causes such as cerebral palsy (CP) with a clear history of brain insult, malformation of cortical development, or brain tumors. A cohort of 31 patients from 30 families (patient 6 and 7 are siblings) was finally recruited into our present study.

### Genetic analyses

Genomic DNA were extracted from peripheral blood using Flexigene DNA Kit (Qiagen GmbH, Germany). Quality of genomic DNA was evaluated by agarose gel analysis and quantity was measured by Qubit® dsDNA assay (Thermo Fisher Scientific, Waltham, MA).

WES was performed in Genome Diagnostics Nijmegen and our local setting (HKU). WES and the data analysis in Genome Diagnostic Nijmegen were performed as described previously [13]. In our local setting, WES was performed as described in our previous study [34]. Exome libraries preparation and quality control were performed according to the manufacturer instructions. The libraries were sequenced by Illumina HiSeq 1500 or NextSeq 500 sequencing platform with a targeted sequencing coverage of 100x. Data processing has been done by our in-house developed bioinformatics pipeline. Briefly, filtered raw reads were mapped to the reference human genome (GRCh37/hg19) by Burrow-Wheeler Aligner (BWA) 0.7.10. Genome Analysis Toolkit (GATK) best practices v3.4-46 was used for variant calling by HaplotypeCaller and the variants were annotated by Annotate Variation (ANNOVAR). First-tier variant analysis was based on a virtual gene panel consist of 272 movement disorder-related genes (Additional file 1: Table 1), 244 mitochondrial disease-related genes (Additional file 1: Table 2) and genes in MitoCarta 2.0 encoding protein with strong support of mitochondrial localization (http://www.broadinstitute.org/pubs/MitoCarta). Even though patients with clinical suspicion of a mitochondrial disorders were excluded, the analysis also included mitochondrial-related genes as the phenotypic presentation for both movement and mitochondrial disorders are highly overlapping. If pathogenic variant(s) could not be identified in these genes, open-exome analysis will be performed. Some of the data was jointly analysis by collaborators at Yale University. Raw WES data from HKU was transferred. Reads were aligned to hg19 reference genome using bwa-mem and processed according to the GATK best practice guidelines. Copy-number variants (CNV) were called using gcnv (part of GATK4). The variants were annotated using Variant Effect Predictor through Hail and then uploaded to seqr (https://seqr.broadinstitute.org/) for analysis. Rare variants were assessed for pathogenicity based on the American College of Medical Genetics (ACMG) guideline [35]. Potential disease-causing variants and segregation analysis were performed by Sanger sequencing.

## Supplementary information

**Additional file 1: Supplementary table 1:** 272 movement disorder-related genes; **Supplementary table 2:** 244 mitochondrial disease-related genes; **Supplementary table 3:** Clinical features of patients with no genetic variant found.

## Data Availability

The datasets used and/or analysed during the current study are available from the corresponding author on reasonable request.

## Abbreviations

MDs: Movement disorders
NGS: Next-generation sequencing
WES: Whole exome sequencing
SPG: Spastic paraplegia
MRI: Magnetic resonance imaging
VUS: Variants of uncertain significance
GPi-DBS: Globus pallidus interna deep brain stimulation
ATP: Adenosine-5′-triphosphate
AHC: Alternating hemiplegia of childhood
KD: Ketogenic diet
