# Supplementary material for: Exome sequencing in paediatric patients with movement disorders
Source: Orphanet J Rare Dis. 2021 Jan 15;16:32. doi: 10.1186/s13023-021-01688-6 (PMC7809769; doi:10.1186/s13023-021-01688-6)
Supplement: Supplementary file 1 — Additional file 1: Supplementary table 1: 272 movement disorder-related genes; Supplementary table 2: 244 mitochondrial disease-related genes; Supplementary table 3: Clinical features of patients with no genetic variant found. [file 13023_2021_1688_MOESM1_ESM.docx]

**Supplementary table 1:**

**272 movement disorder-related genes**

| *ABCB7* | *ATXN8OS* | *EIF2B2* | *JPH3* | *PCCB* |
| --- | --- | --- | --- | --- |
| *ABCD1* | *B4GALNT* | *EIF2B3* | *KCNA1* | *PDE10A* |
| *ABHD12* | *BCAP31* | *EIF2B4* | *KCNC3* | *PDE8B* |
| *ACAT1* | *BCKDHA* | *EIF2B5* | *KCND3* | *PDGFB* |
| *ACP33* | *BCKDHB* | *EIF4G1* | *KCNJ10* | *PDGFRB* |
| *ACTB* | *BEAN1* | *ELOVL5* | *KCNMA1* | *PDHA1* |
| *ADCK3* | *BICD2* | *ERLIN1* | *KCTD17* | *PDHX* |
| *ADCY5* | *BSCL2* | *ERLIN2* | *KIAA0196* | *PDSS1* |
| *AFG3L2* | *C10orf2* | *FA2H* | *KIAA0226* | *PDSS2* |
| *ALDH3A2* | *C12orf65* | *FAR1* | *KIAA0415* | *PDYN* |
| *ALS2* | *C19orf12* | *FBXO7* | *KIAA1840* | *PEX7* |
| *ALSIN* | *CA8* | *FGF14* | *KIF1A* | *PEX10* |
| *ANO10* | *CACNA1A* | *FLVCR1* | *KIF1C* | *PHYH* |
| *ANO3* | *CACNA1G* | *FTL* | *KIF5A* | *PIK3R5* |
| *AP4B* | *CACNB4* | *FUS* | *KMT2B* | *PINK1* |
| *AP4B1* | *CCT5* | *GALC* | *L1CAM* | *PLA2G6* |
| *AP4E1* | *CHCHD2* | *GAN* | *LRRK2* | *PLEKHG4* |
| *AP4M1* | *CIZ1* | *GBA* | *MARS2* | *PLP1* |
| *AP4S1* | *COASY* | *GBA2* | *MECP2* | *PMM2* |
| *AP5Z1* | *COL6A3* | *GCDH* | *MICU1* | *PNKD* |
| *APAM1* | *COQ2* | *GCH1* | *MRE11A* | *PNKP* |
| *APTX* | *COQ9* | *GFAP* | *MMADHC* | *PNPLA6* |
| *ARSA* | *CP* | *GJC2* | *MTHFR* | *POLG* |
| *ARX* | *CSTB* | *GLB1* | *MTPAP* | *POLR3A* |
| *ASPA* | *CYP27A1* | *GLDC* | *MTTP* | *POLR3B* |
| *ATCAY* | *CYP2U1* | *GNAL* | *MUT* | *PPP2R2B* |
| *ATL1* | *CYP7B1* | *GNAO1* | *NIPA1* | *PRKCG* |
| *ATM* | *DBT* | *GOSR2* | *NKX2-1* | *PRKRA* |
| *ATN1* | *DCAF17* | *GPR56* | *NOL3* | *PRNP* |
| *ATP1A2* | *DCTN1* | *GRID2* | *NOP56* | *PRRT2* |
| *ATP1A3* | *DDC* | *GRM1* | *NPC1* | *PSEN1* |
| *ATP13A2* | *DDHD1* | *HEXB* | *NPC2* | *PTS* |
| *ATP2B3* | *DDHD2* | *HPCA* | *NT* | *QDPR* |
| *ATP6AP2* | *DJ-1 (PARK7)* | *HPRT* | *NT5C2* | *RAB39B* |
| *ATP7B* | *DLAT* | *HPRT1* | *NUP62* | *REEP1* |
| *ATXN1* | *DLD* | *HSPD1* | *OPA1* | *RNASEH2A* |
| *ATXN10* | *DNAJC13* | *HTRA2* | *PANK2* | *RNASEH2B* |
| *ATXN2* | *DNAJC6* | *HTT* | *Parkin (PARK2)* | *RNASEH2C* |
| *ATXN3* | *DNMT1* | *ISG15* | *PAX6* | *RNF170* |
| *ATXN7* | *EIF2B1* | *ITPR1* | *PCCA* | *RTN2* |
| *SACS* | *SLC25A15* | *SPR* | *TIMM8A* | *WDR45* |
| *SACSIN* | *SLC30A10* | *SPR3* | *TMEM240* | *WDR81* |
| *SAMHD1* | *SLC33A1* | *SPTBN2* | *TMEM67* | *WWOX* |
| *SCN4A* | *SLC52A2* | *STUB1* | *TOR1A* | *XK* |
| *SCN8A* | *SLC52A3* | *SUCLA2* | *TREX1* | *XPR1* |
| *SERAC1* | *SLC6A3* | *SUOX* | *TTBK2* | *ZFYVE26* |
| *SETX* | *SLC9A1* | *SYNE1* | *TTC19* | *ZFYVE27* |
| *SGCE* | *SMPD1* | *SYNJ1* | *TTPA* | *ZNF592* |
| *SIL1* | *SNCA* | *TAF1* | *TUBB4A* |  |
| *SLC1A3* | *SNX14* | *TBP* | *VAMP1* |  |
| *SLC12A6* | *SPARTIN* | *TDP1* | *VCP* |  |
| *SLC16A2* | *SPAST* | *TECPR2* | *VLDLR* |  |
| *SLC18A2* | *SPG11* | *TENM4* | *VPS13A* |  |
| *SLC19A3* | *SPG20* | *TGM6* | *VPS35* |  |
| *SLC2A1* | *SPG21* | *TH* | *VPS37A* |  |
| *SLC20A2* | *SPG7* | *THAP1* | *VRK1* |  |

**Supplementary table 2:**

**244 mitochondrial disease-related genes**

| *AARS2* | *COX10* | *GDAP1* | *MRPL3* | *OPA1* |
| --- | --- | --- | --- | --- |
| *ABCB7* | *COX14* | *GFER* | *MRPL44* | *OPA3* |
| *ACAD9* | *COX15* | *GFM1* | *MRPS16* | *OXCT1* |
| *ACADM* | *COX20* | *GLRX5* | *MRPS22* | *PANK2* |
| *ACADS* | *COX4I2* | *GTPBP3* | *MRPS23* | *PARS2* |
| *ACADSB* | *COX6A1* | *HADH* | *MRPS7* | *PC* |
| *ACADVL* | *COX6B1* | *HADHA* | *MTFMT* | *PDHA1* |
| *ACAT1* | *COX7B* | *HADHB* | *MTPAP* | *PDHB* |
| *ACO2* | *CPT1A* | *HARS2* | *MTO1* | *PDHX* |
| *ADCK3* | *CPT2* | *HCCS* | *NADK2* | *PDK3* |
| *ADCK4* | *CYC1* | *HIBCH* | *NARS2* | *PDP1* |
| *AFG3L2* | *CYCS* | *HLCS* | *NDUFA1* | *PDSS1* |
| *AGK* | *CYP2U1* | *HMGCL* | *NDUFA2* | *PDSS2* |
| *AIFM1* | *D2HGDH* | *HMGCS2* | *NDUFA4* | *PET100* |
| *APOPT1* | *DARS2* | *HSD17B10* | *NDUFA9* | *PITRM1* |
| *ATP5A1* | *DCAF17* | *HSPA9* | *NDUFA10* | *PMPCA* |
| *ATP5E* | *DDHD1* | *HSPD1* | *NDUFA11* | *PNPLA4* |
| *ATPAF2* | *DDHD2* | *HTT* | *NDUFA12* | *PNPLA8* |
| *BCS1L* | *DGUOK* | *IARS2* | *NDUFA13* | *PNPT1* |
| *BOLA3* | *DLAT* | *IBA57* | *NDUFAF1* | *POLG* |
| *BTD* | *DLD* | *IDH2* | *NDUFAF2* | *POLG2* |
| *C10orf2* | *DNA2* | *IDH3B* | *NDUFAF3* | *PPOX* |
| *C12orf65* | *DNAJC19* | *ISCA2* | *NDUFAF4* | *PUS1* |
| *C20orf7* | *DNM1L* | *ISCU* | *NDUFAF5* | *QRSL1* |
| *COA3* | *EARS2* | *KARS* | *NDUFAF6* | *RARS2* |
| *COQ7* | *ECHS1* | *L2HGDH* | *NDUFB3* | *RMND1* |
| *CA5A* | *ELAC2* | *LARS2* | *NDUFB9* | *RNASEH1* |
| *CARS2* | *ETFA* | *LIAS* | *NDUFB11* | *RRM2B* |
| *CEP89* | *ETFB* | *LIPT1* | *NDUFS1* | *RTN4IP1* |
| *CHCHD10* | *ETFDH* | *LONP1* | *NDUFS2* | *SAMHD1* |
| *CHKB* | *ETHE1* | *LRPPRC* | *NDUFS3* | *SARS2* |
| *CLPB* | *FARS2* | *LYRM4* | *NDUFS4* | *SCO1* |
| *CLPP* | *FASTKD2* | *LYRM7* | *NDUFS6* | *SCO2* |
| *COA5* | *FBXL4* | *MARS2* | *NDUFS7* | *SDHA* |
| *COA6* | *FDX1L* | *MFF* | *NDUFS8* | *SDHAF1* |
| *COASY* | *FH* | *MFN2* | *NDUFV1* | *SDHAF2* |
| *COQ2* | *FLAD1* | *MGME1* | *NDUFV2* | *SDHB* |
| *COQ4* | *FOXRED1* | *MPC1* | *NFU1* | *SDHC* |
| *COQ6* | *FXN* | *MPV17* | *NFS1* | *SDHD* |
| *COQ9* | *GARS* | *MRPL12* | *NUBPL* | *SERAC1* |
| *SFXN4* | *SLC25A4* | *TARS2* | *TRMU* | *UQCRC2* |
| *SLC19A3* | *SLC25A42* | *TAZ* | *TRNT1* | *VARS2* |
| *SLC22A5* | *SLC25A46* | *TBCE* | *TSFM* | *XPNPEP3* |
| *SLC25A1* | *SPG7* | *TIMM8A* | *TTC19* | *YARS2* |
| *SLC25A12* | *STAT2* | *TK2* | *TUFM* |  |
| *SLC25A19* | *SUCLA2* | *TMEM126A* | *TYMP* |  |
| *SLC25A20* | *SUCLG1* | *TMEM70* | *UQCC2* |  |
| *SLC25A26* | *SURF1* | *TPK1* | *UQCC3* |  |
| *SLC25A3* | *TACO1* | *TRIT1* | *UQCRB* |  |
| *SLC25A38* | *TANGO2* | *TRMT5* | *UQCRQ* |  |

**Supplementary table 3:**

**Clinical features of patients with no genetic variant found**

| Patient | Sex | Ethnicity | Movement disorders | Onset | Dysmorphic features | Congenital anomalies | Other clinical features | MRI features | aCGH findings |
| --- | --- | --- | --- | --- | --- | --- | --- | --- | --- |
| 2 | F | African Chinese | Dystonia, Spasticity | Birth | - | Duodenal atresia | left eye cataract, Bilateral hearing impairment, Severe ID, right eye dysgenesis | Progressive cerebral atrophy, bilateral basal ganglia T2 hyperintensity | Normal |
| 3 | M | Chinese | Dystonia with status dystonicus, Spasticity | Birth | + | - | Severe ID, Retinopathy of prematurity, Bilateral inguinal hernia, oromotor dysfunction, extreme prematurity | Periventricular leukomalacia with corpus callosum thinning, cerebral and cerebellar atrophy | Not done |
| 4 | M | Chinese | Dystonia | 4y | - | - | Mild ID | Iron deposit at bilateral globus pallidus and anterior midbrain | Not done |
| 5 | M | Chinese | Spastic paraplegia | 1y | + | - | Mild upper limb weakness, amyotrophy at distal lower limbs, Mild ID | Normal | Not done |
| 9 | F | Chinese | Spastic paraplegia with upper limb involvement | Infancy | - | - | - | Normal | Not done |
| 10 | M | Chinese | Cerebellar ataxia, Dystonia | 14y | + | - | Short statue, failure to thrive, Mild ID to Limited intelligence | Normal | Not done |
| 11 | F | Chinese | Spastic paraplegia | Infancy | + | - | Moderate ID , neuromuscular involvement | Normal | Not done |
| 13 | F | Chinese | Dystonia, Rigidity, Spasticity | Infancy | - | - | Global delay | Normal | Not done |
| 15 | M | Chinese | Dystonia, Spasticity | Infancy | - | - | Prematurity with stormy neonatal events, downward gaze palsy, Global delay, bilateral hearing impairment, | Bilateral basal ganglia T2 hyperintensity, Cerebral atrophy | Not done |
| 16 | M | Chinese | Cerebellar ataxia, Spasticity, Dystonia | 2y | + | - | Myopathy, Neurogenic bladder | Iron deposit at bilateral globus pallidus, substantia nigra, red nucleus; Leukodystrophy; Progressive cerebral and spinal cord atrophy | Not done |
| 18 | M | Chinese | Dystonia | Birth | - | - | Global delay, oromotor dysfunction | Bilateral basal ganglia and thalami T2 hyperintensity | Normal |
| 21 | F | Pakistani | Dystonia, Ataxia, Paroxysmal dyskinesia | Infancy | - | - | Non paralytic strabismus, slow saccadic movements, Mild to moderate ID | Normal | Not done |
| 22 | M | Pakistani | Dystonia, Ataxia, Paroxysmal dyskinesia | Infancy | - | - | Non paralytic strabismus, slow saccadic movements, global delay | Normal | Not done |
| 24 | M | Chinese | Paroxysmal dyskinesia | 3y | - | - | Attention deficit hyperactivity disorder | Normal | Not done |
| 25 | F | Chinese | Dystonia, Spasticity | 2y | - | - | - | Normal | Not done |
| 26 | M | Chinese | Cerebellar ataxia, spasticity | 10y | - | - | Attention deficit hyperactivity disorder, autism spectrum disorder, bilateral hearing impairment | Normal | Not done |
| 28 | M | Chinese | Cerebellar ataxia, chorea | Early childhood | - | - | Attention deficit, hyperactivity disorder, right eye ptosis | Normal | Not done |

MRI = Magnetic Resonance Imaging; y = years; ID = intellectual disability
